# Next Generation Non-Vacuum, Maskless, Low Temperature Nanoparticle Ink Laser Digital Direct Metal Patterning for a Large Area Flexible Electronics

**DOI:** 10.1371/journal.pone.0042315

**Published:** 2012-08-10

**Authors:** Junyeob Yeo, Sukjoon Hong, Daehoo Lee, Nico Hotz, Ming-Tsang Lee, Costas P. Grigoropoulos, Seung Hwan Ko

**Affiliations:** 1 Applied Nano Technology and Science Lab, Department of Mechanical Engineering, Korea Advanced Institute of Science and Technology, Daejeon, Korea; 2 Laser Thermal Lab, University of California, Berkeley, California, United States of America; 3 Duke University, Department of Mechanical Engineering and Materials Science, Thermodynamics and Sustainable Energy Lab, Durham, North Carolina, United States of America; 4 Department of Mechanical Engineering, National Chung Hsing University, Taichung, Taiwan R.O.C.; Texas A&M University, United States of America

## Abstract

Flexible electronics opened a new class of future electronics. The foldable, light and durable nature of flexible electronics allows vast flexibility in applications such as display, energy devices and mobile electronics. Even though conventional electronics fabrication methods are well developed for rigid substrates, direct application or slight modification of conventional processes for flexible electronics fabrication cannot work. The future flexible electronics fabrication requires totally new low-temperature process development optimized for flexible substrate and it should be based on new material too. Here we present a simple approach to developing a flexible electronics fabrication without using conventional vacuum deposition and photolithography. We found that direct metal patterning based on laser-induced local melting of metal nanoparticle ink is a promising low-temperature alternative to vacuum deposition– and photolithography-based conventional metal patterning processes. The “digital” nature of the proposed direct metal patterning process removes the need for expensive photomask and allows easy design modification and short turnaround time. This new process can be extremely useful for current small-volume, large-variety manufacturing paradigms. Besides, simple, scalable, fast and low-temperature processes can lead to cost-effective fabrication methods on a large-area polymer substrate. The developed process was successfully applied to demonstrate high-quality Ag patterning (2.1 µΩ·cm) and high-performance flexible organic field effect transistor arrays.

## Introduction

The development of electric circuit fabrication on a flexible polymer substrate has gained significant interest as a pathway to low cost or large area electronics [1]–[18]. The conventional vacuum metal deposition and photolithographic patterning methods are well developed for Silicon based microelectronics. However, flexible polymer substrates are chemically incompatible with corrosive resists, etchants and developers used in conventional IC (integrated circuit) processing. In practice, conventional IC fabrication processes are subject to limitations, in that they are multi-step, involve high processing temperatures, toxic waste and are therefore expensive [19]. Furthermore, the increasing size of electronic devices such as displays or solar cells poses great difficulty in adapting standard microfabrication processes, including highly complex steps, slow processing time and astronomical cost increase especially due to expensive vacuum chamber and photo-mask [19]. Furthermore, it is almost impossible to change the design of the expensive photomask once it is fabricated. Therefore, as an alternative to conventional vacuum deposition and photolithographic metal patterning approach, there is a strong need for a development of a fully *“digital”* maskless, high resolution, low temperature, and fast metal patterning method on a flexible polymer substrate to realize cost-effective flexible electronics [19]–[20].

For these reasons, the development of alternative maskless, direct digital, high resolution patterning techniques to fabricate conductive micro/nano-patterns at the atmospheric pressure and low temperature without using any vacuum deposition or photolithography has attracted wide attention in recent years. One of the most promising alternatives is the direct patterning of solution-deposited metal nanoparticles (NPs). The development of metal NP solution ink enabled (a) inexpensive solution-based metal deposition approach without using expensive vacuum deposition and (b) low temperature metal deposition process which allows using heat-sensitive, cheap polymer as a substrate. Examples of NP ink-based direct metal patterning include screen printing [21]–[22], micro-contact printing [23]–[24], aerosol jet deposition and inkjet printing [25]–[26]. These processes can fabricate conductive micro-patterns onto the substrate in one-step. Solution-deposited and directly patterned metal NPs can then be transformed into continuous metal patterns through a low temperature thermal sintering process. Due to the thermodynamic size effect, the melting temperature of the metal NPs can be reduced to the range of 100–200°C [27]–[28]. However, the resolution those NP ink based direct metal patterning approaches was still limited to few tens of microns and the flexible substrate suffers from thermal degradation or mechanical wrapping during the several hours long bulk heating to induce NP melting [21]–[26].

In this paper, as a potential alternative to the conventional vacuum deposition and photo-lithography based metal patterning, we introduce a novel digital, low-temperature, fast, high resolution direct metal patterning method wherein solution-deposited metal NPs were selectively sintered by applying a raster scanning focused laser to pattern metal electrode on a polymer substrate in a single step for the fabrication of flexible electronics. The proposed laser based digital direct metal patterning method can produce high resolution (down to a couple of microns resolution) metal patterning on a large polymer substrate (over 4 inch wafer) in a very fast single step (fraction of minutes) at room temperature in an ambient pressure. Moreover, the *“digital”* nature of our process can remove the need for expensive photo-masks and allows easy design change. To demonstrate the feasibility of our approach for high performance flexible electronics, we demonstrated large area OFET arrays on a flexible substrate. All of these characteristics are expected to greatly contribute to the development for next generation cost-effective metal patterning to realize flexible electronics.

## Materials and Methods

### Ag nanoparticle preparation and characterization

The Ag NP synthesis were modified from a two-phase reduction method reported by Korgel *et al* [29]. Aqueous silver ions (AgNO_3_) were mixed in a toluene solution containing long-chain alkylammonium surfactants to form a two-phase system. 0.2 M tetroactylammonium bromide ((C_8_H_17_)_4_NBr) was mixed with 20.4 mL of toluene and added to 30 mM AgNO_3_ in 30 mL of deionized (DI) water. 1 hr vigorous stirring transferred the silver ions (Ag+) into the organic phase (toluene) and the aqueous phase was removed. A measured quantity (0.16 mg) of capping agent, a long-chain thiol (dodecanethiol (C_12_H_25_SH)), was added to the silver ion (Ag+) solution while stirring. After the dodecanethiol/Ag+ solution was stirred for 15 min, a reducing agent, 0.43 M aqueous sodium borohydride (NaBH_4_), mixed in 24 mL of DI water was added into the organic phase with a fast addition over approximately 10 s to nucleate nano-crystals. The thiol stabilizes the growing colloids by binding to the nanocrystal surface and helps to maintain a relatively narrow particle size distribution. The mixture reacted at room temperature for three and half hours. Chloroform was removed with a rotary evaporator and the leftover black particles suspended in ethanol and sonicated briefly. The particles were washed with ethanol and acetone to remove the phase transfer catalyst, excess thiol, and reaction byproducts and air dried. Monolayer-protected Ag nanoparticles were suspended in organic solvent (toluene or alpha-terpineol) with 10% in weight.

**Figure 1:**
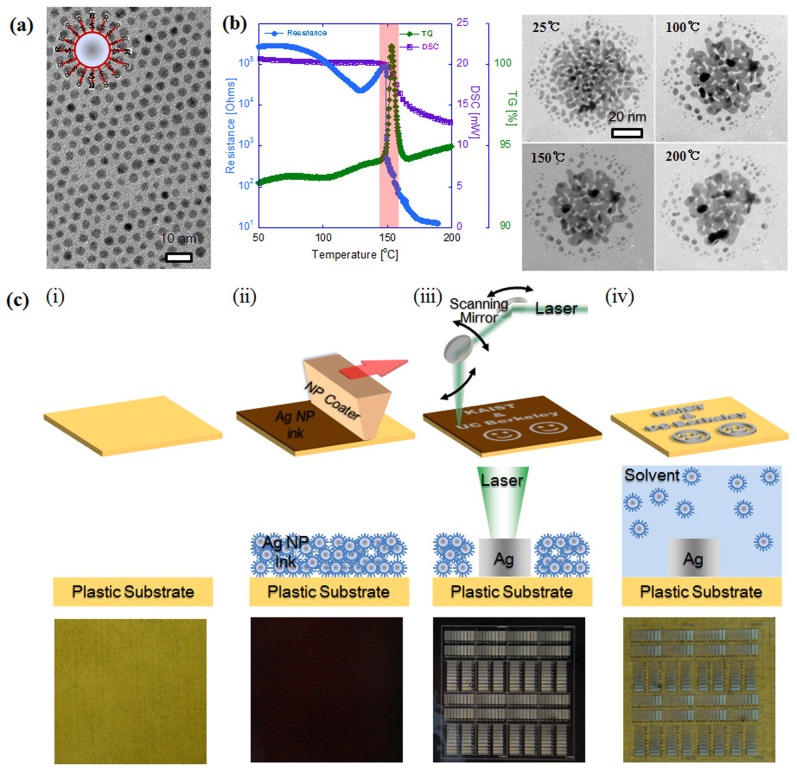
Digital Direct Metal Patterning (DDMP) Process on a Flexible Substrate at Room Temperature and Atmospheric Pressure without Using Any Vacuum Deposition and Photolithography Processes. **a,** TEM image of SAM coated Ag NPs synthesized via two phase method. Inset picture shows the schematics of the SAM coated Ag NP. **b,** Melting characteristics of the Ag NPs at various heating temperature; resistance change (blue), thermo gravimetric analysis (green) and differential scanning calorimetry (purple) in left graph, and TEM images (right). **c,** DDMP process schematics; (i) a polymer substrate preparation, (ii) Ag NP ink coating on a polymer substrate, (iii) laser scanning and local melting of the printed Ag NP ink film, (iv) washing out non-laser-processed Ag NPs to leave laser-processed Ag patternings on a polymer substrate. Middle row shows the cross section and bottom row shows the top view.

The size of synthesized nanoparticles was distributed from 3 to 6 nm as measured by TEM (JEM-2100F HR) (figure 1a). The size was coarsely tunable by adjusting the ratio of capping groups to silver ions, whereas size selective precipitation was employed to narrow the initial size distribution. The thermal characteristics of the Ag nanoparticles were measured by DSC (SETSYS 16/18) and TGA (SETSYS 16/18) (figure 1b) with 10°C/min scanning rate for the first heating.

### Semiconducting polymer synthesis

All chemicals were purchased from Aldrich and used without further purification unless otherwise noted. All solvents were purified on a solvent purification system. All reactions were performed under N_2_ unless otherwise noted. All extracts were dried over anhydrous MgSO_4_ and solvents were removed by rotary evaporation with vacuum assist. Flash chromatography was performed using Merck Kieselgel60 (230–400 mesh) silica. 1H NMR spectra were recorded with Brüker AMX-300, AM-400 or DRX-500 instruments using CDCl_3_ as the solvent unless otherwise noted. Analytical size exclusion chromatography (SEC) in THF was performed at 35°C at a nominal flow rate of a 1.0 mL/min on a chromatography line calibrated with linear polystyrene standards (162–2,100,000 Da) and fitted with three 7.5×300 mm PL gel columns (5 µm particle size). The columns have a pore size of 105, 103, and 500 Å, respectively. The SEC system consists of a Waters 510 pump, a Waters 717 autosampler, and a Waters 486 UV-Vis detector detecting at 254 and 450 nm. Polymer solutions were prepared by adding 100 µL of a 1 mg/mL solution of the polymer in *o*-DCB to 1 mL of THF, then filtering through 0.2 µm pore size PVDF filters (Whatman) before injection [30]. Further information on semiconducting polymer characterization can be found in the Supporting Information (figure S4).

### Digital Direct Metal Patterning (DDMP) process

After coating Ag NP ink on the substrate, continuous wave green wavelength lasers (Nd:YAG-532 nm, Ar ion-514.5 nm) were scanned by 2D galvanometric scanning mirror system (SCANLAB, hyrrySCAN II) to raster scan focused laser spot and induce local Ag NP melting. The laser scanner system was controlled by computer with CAD software (SCAPS GmbH, SAMLight) to draw arbitrary 2D images. The laser scanning speed and laser power were adjusted in 0∼3 m/s and 0∼400 mW depending on the substrate.

### OFET fabrication and characterization

The OFET has a typical bottom gate/bottom contact coplanar transistor configuration. First, rectangular shape gate electrode (500 µm×110 µm) was patterned on a PI substrate by DDMP process. Then, as a dielectric layer, PVP (poly-4-vinylphenol, MW ∼8,000 AMU) dissolved in hexanol with a small amount of the cross-linking agent (poly (melamine-co-formaldehyde)), was spincoated on top of the gate line and cross-linked at 150°C (150 nm thick). Source and drain electrodes were patterned on PVP dielectric layer by DDMP process. As a last step, the air stable carboxylate-functionalized polythiophene semiconducting polymer was spincoated on source and drain electrodes and annealed at 120°C on a hot plate for 3 minutes. The OFET output and transfer characteristics were characterized using HP4155A semiconductor parameter analyzer and a probe station with the micro-positioning manipulators in a dark Faraday cage in air. For output characteristics measurement, the drain voltage (V_d_) was scanned from 0 to −70 V and the drain current (I_d_) was measured while gate voltage (V_g_) was fixed at −70,−50,−30,−10,10 V during each V_d_ scanning. For transfer characteristics measurement, the gate voltage (V_g_) was scanned from 10 to −70 V and the drain current (I_d_) was measured while drain voltage (V_d_) was fixed at −70 V.

## Results and Discussion

To overcome the limitations in conventional photolithography and vacuum metal deposition, our newly developed Digital Direct Metal Patterning (DDMP) process exploits the novel properties of nanomaterials due to their dramatically larger surface to volume ratio than bulk materials. Especially, three novel properties of nanoparticles (NPs) play very important roles in DDMP process development: (1) unique thermodynamic property such as size dependent melting temperature drop and low thermal diffusivity for deposited NP films [28], (2) unique optical properties such as strong light absorption that exhibits distinct peaks at resonance frequencies in the visible range [20], and (3) easy formation of nanoparticle ink.

Firstly, size dependent melting temperature drop of NPs allows the development of plastic compatible low temperature metal deposition process. The size of the Ag NPs applied in this research was distributed in the range of 3∼6 nm as measured by transmission electron microscopy (TEM) (figure 1a) and they were prepared using a two-phase reduction method [29] whereby the NPs were encapsulated by dodecanethiol (C_12_H_25_SH) self-assembled monolayer (SAM) to ensure the long-term stability of the Ag NP solution. Due to the presence of SAM on NP surface (figure 1a, inset), metallic NPs are not directly touching each other as shown in TEM picture (figure 1a), thus Ag NP film composed of discrete Ag NPs shows poor electrical conductance. To form highly conductive continuous metal, discrete Ag NPs need to be thermally melted. The synthesized Ag NPs showed evidence of melting behavior at significantly low temperatures (∼150°C) compared to the melting temperature of bulk Ag (960°C) due to thermodynamic size effect [28], as indicated by the dramatic change in the thermo gravimetric analysis (TGA), differential scanning calorimetry (DSC), electrical resistance measurement during NP melting illustrated in figure 1b (red box). In-situ direct TEM observation (figure 1b right pictures) of NP melting microscopic behavior at various heating temperature revealed how individual NPs started to melt and formed electrically conductive path. Secondly, Ag NPs shows strong absorption peak in green wavelength due to surface plasmon oscillation mode of conduction electrons [31]. This allows the possibility of using laser as a local heat source to locally induce Ag NP melting to form conductive metal patterns. Local laser heating is advantageous due to reduced thermal damage to the substrate [19], and enhance the process resolution with highly confined and efficient local energy deposition and NP melting. Compared with inkjet printed Ag NP pattern whose resolution is limited to few tens of microns due to nozzle size limitation, DDMP process can demonstrate even sub-micron metal patterns [32]. Thirdly, NPs can easily form nano-inks to be applied for cost-effective printed electronics process development. The development of metal NP solution ink enabled inexpensive solution-based metal printing approaches without using expensive vacuum deposition and also allowed low temperature metal deposition process which allows using heat-sensitive, cheap polymer as a substrate. Solution processible metal deposition and patterning is the core technology to realize ultra-low cost printed electronics development such as roll-to-roll printing.

DDMP process combines the Ag NP ink coating and subsequent selective laser local melting as shown in figure 1c. The Ag NP ink was coated onto a polymer substrate (figure 1c(i)) to form NP thin film by spin casting or slot die coating (figure 1c(ii)). The prepared Ag NP film was selectively melted by scanning continuous wave (CW) focused Ar ion (514.5 nm wavelength) or Nd:YAG (532 nm wavelength) laser beam with a 2D laser scanner system connected to the CAD (computer aided design) software operated computer to draw arbitrary patterns (figure 1c(iii)). Upon completion of the laser scanning process, the remaining non-melted and un-laser-processed Ag NPs were simply washed away in the organic solvent to leave laser processed metal nano/micro patterns (figure 1c(iv)). During the DDMP process, sometimes fume was observed due to SAM decomposition and it was removed from the laser spot by suction. Detailed information on DDMP system can be found in figure S1, figure 1c(iv) bottom picture shows 5500 Ag line patterns on PI (polyimide) substrate. It should be noted that the metal patterning process was very fast (fraction of tens of seconds to minutes) and the pattern design can be easily modified by simply changing the CAD data. In comparison, for conventional metal patterning process, pattern design change requires an entirely new photo-mask. The fast metal patterning process was possible due to unique characteristics of Ag NPs. The general laser power requirement for Ag NP melting is very small (10∼350 mW) due to size dependent melting temperature drop and highly efficient laser absorption. Therefore, low laser energy requirement enables fast laser scanning speed over 1 m/s, thus allows very fast metal patterning process. Besides, the process is single step and there is no need for vacuum environment thus removes the long process time to reach high vacuum, which enables very fast metal patterning process development.

Combined with the efficient laser absorption of the Ag NPs, laser as a local heat source can greatly minimize the thermal damage to the substrate. The integrity of the plastic substrate was check after the DDMP process and removal of Ag pattern from the substrate. The inspection confirmed that there was no noticeable plastic substrate damage in the proper laser power range. The adhesion of the Ag pattern on the plastic substrate is another important factor in successful flexible electronics fabrication and it was strong enough to survive the tape test.

**Figure 2:**
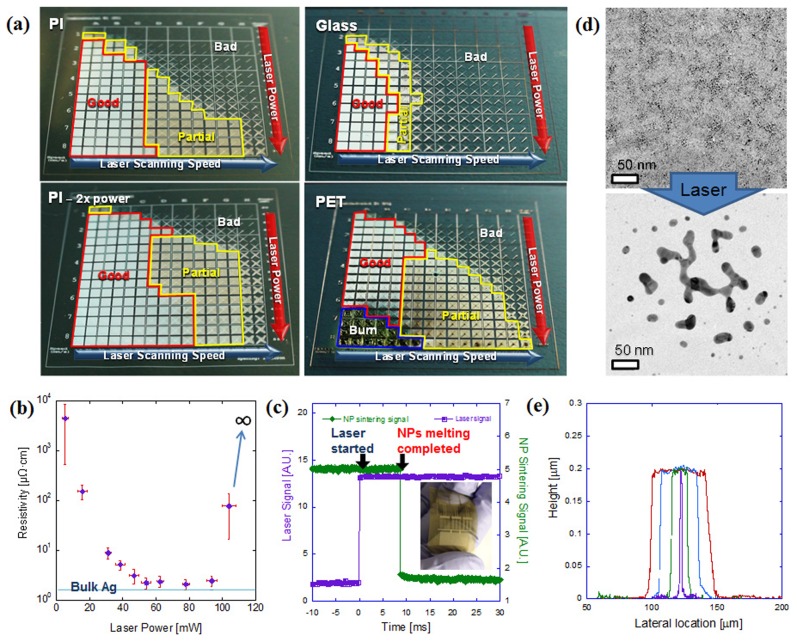
Process Characterization of Laser Induced Melting of Ag NP Ink During DDMP. **a,** Combinatorial study for optimum laser power *vs*. laser scanning speed on PI (left top & bottom), glass (right top) PET(right bottom). Laser power and laser scanning speed in “*Good*” regimes (red boxes) were used for real process. Detailed laser scanning speed and laser power values can be found in Supporting Information (figure S2). The total size and unit box size for combinatorial study was 2 cm×2 cm and 1 mm×1 mm respectively. **b,** Resistivity change at various laser power. Blue line shows the resistivity value of bulk Ag (1.59 µΩ·cm) **c,** Process time characterization by transient resistance change measurement; time required for Ag NP melting (green) after laser irradiation (purple). Inset picture shows the Ag electrode patterns for process time measurement on a polymer substrate. **d,** TEM pictures of Ag NPs before (top) and after (bottom) laser irradiation. **e,** AFM cross sectional profiles of laser process Ag line patterns.

Arbitrary metal patterns can be achieved by raster scanning of the focused laser beam with CAD data. Successful metal patterning through DDMP process should satisfy both sufficient NP melting and metal pattern's strong adhesion to the substrate. Figure 2a shows the combinatorial study of the laser power and laser scanning speed for successful DDMP process development on PI, PET (Polyethylene terephthalate) and glass substrate (see figure S2 for the detailed laser power and laser scanning speed information of the combinatorial study). When the laser power was low and laser scanning speed high, NPs did not experience melting and were removed from the substrate after washing (“*Bad*” regimes in figure 2a) while the laser will damage the substrate for high laser power and slow laser scanning speed case (“*Burn*” regimes in PET sample, figure 2a). Optimum laser power (“*Good*” regimes in figure 2a) was 10∼160 mW for 0.1∼0.7 m/s scanning speed on PI substrate (figure 2a top left picture), while the metal patterning adhesion was poor or NP melting was incomplete (“*Partial*” regimes in figure 2a) when the laser scanning speed was higher than the optimum speed. When the laser power was doubled (figure 2a bottom left picture), the maximum scanning speed for “*Good*” regimes was extended to 1.0 m/s on the same substrate. Glass substrate showed smaller “*Good*” regime (figure 2a top right picture) at the same laser power range requirement than polymer substrate because glass possess higher thermal conductivity than polymer and the heat loss to the glass substrate is larger than polymer substrate. PET showed “*Burn*” regime (figure 2a bottom right picture) because it has lower melting temperature than PI substrate.

The resistivity of the Ag NP thin film on PET substrate initially drops and then gets saturated, and finally increases dramatically as the applied laser power increases (figure 2b). Low power laser (>40 mW) induces partial melting of Ag NPs and cause the initial resistivity drop from virtually infinite to several tens of µΩ·cm. Moderate laser power (<40 mW, >100 mW) induces full melting of Ag NPs and resistivity shows constant minimum value (2.1 µΩ·cm). High laser power (>100 mW) causes substrate damage (figure 2a, PET) and again dramatic resistivity increase. The achieved minimum resistivity of DDMP processed Ag electrode was only 130% that of bulk Ag (1.59 µΩ·cm). This signifies that the metal patterning from current process can produce high quality metal electrode and can be readily applied for high performance electronics. The discrepancy between the bulk Ag and DDMP processed Ag electrode could be mainly due to the electron scattering caused by the electrode surface roughness and the nano-pores inside the sintered metal conductor generated by the trapped residual organic solvent and SAM remnants.

The speed of the laser induced Ag NP melting limits the whole process speed. Figure 2c shows the characteristic time of the DDMP process. The time lag between laser irradiation (green line) and conductive metal electrode formation (purple line) was measured by transient resistance change during the laser irradiation on Ag NP ink (see figure S3 for detailed experiment information) and was found to be 5∼10 ms. This resistance change represents the NP film transformation into continuous metal thin film through a huge number of NPs. This NP melting characteristic time will be much shorter in a small scale such as the sintering time via melting of a pair of NPs (0.5 ns) reported by Heng *et al.* [33]–[34]. TEM pictures in figure 2d show nanoscopic view on how SAM protected discrete Ag NPs melt, sinter and form connected conductive path upon exposure to laser irradiation.

The resolution achieved by this process is defined by the focused laser beam diameter and applied laser power. The smallest line width achievable was 2 µm (figure 2e, purple line) by single scanning of tightly focused laser beam, while repeated laser scanning and looser focusing can realize lines as wide as needed.

**Figure 3:**
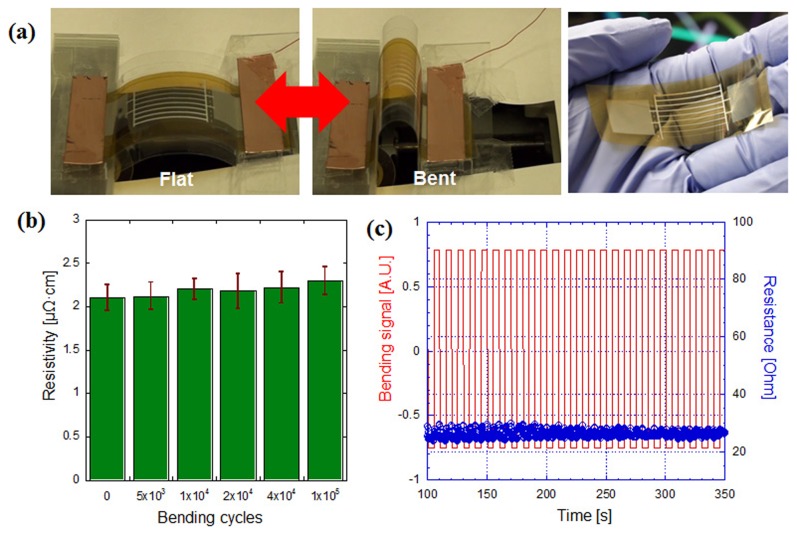
Electrical and Mechanical Characterization of the Flexible Electrode Patterns Prepared by DDMP Process the Under Cyclic Bending. **a,** Digital images of cyclic bending test at flat (left) and bent (right) state. Right picture shows the flexible electrode sample for cyclic bending test. **b,** Resistivity measurement of the DDMP processed metal electrode after 5∼100 k bending cycles. Note that no significant resistivity change was observed after 100,000 times bending. **c,** Transient resistance change of the DDMP processed metal electrode during the cyclic bending.

Flexible electronics on a polymer substrate are subject to various mechanical deformation conditions including as bending, compression and tension. Mechanical strains may degrade the structural and the electrical performance of flexible electronics. To check the electrical reliability of the DDMP processed metal electrodes on a polymer substrate, electrical and structural characterization under cyclic bending deformation was carried out. Figure 3a shows the experimental setup for the cyclic bending deformation of DDMP processed Ag micro lines on a PI substrate (figure 3a right picture). With one fixed end and moving the other end in a cyclic motion, over 100,000 bending deformation cycles with bending radius of curvature between 5 mm (figure 3a, “*Bent*” case) and 300 mm (figure 3a, “*Flat*” case) were applied. The electrical resistivity of the DDMP processed Ag microwires in 10–100 µm width and 3 cm length (figure 3a right picture) was measured for different bending cycles (0, 5 k, 10 k, 20 k, 40 k, 100 k cycles). As shown in cyclic bending test (figure 3b), resistivity values did not show noticeable changes through the course of bending deformation cycles within error range. Besides, in-situ transient bending test showed small resistance change during the each bending cycles (figure 3c, see Movie clip S1 for cyclic bending test). In terms of microscopic structural change, the SEM images confirmed that the DDMP processed Ag microstructures maintained the same surface morphology with no observable development of micro/nanoscale cracks or change of surface roughness after 100,000 bending cycles. This verifies that the DDMP processed metal patterning can be readily used for high performance flexible electronics with high reliability.

**Figure 4:**
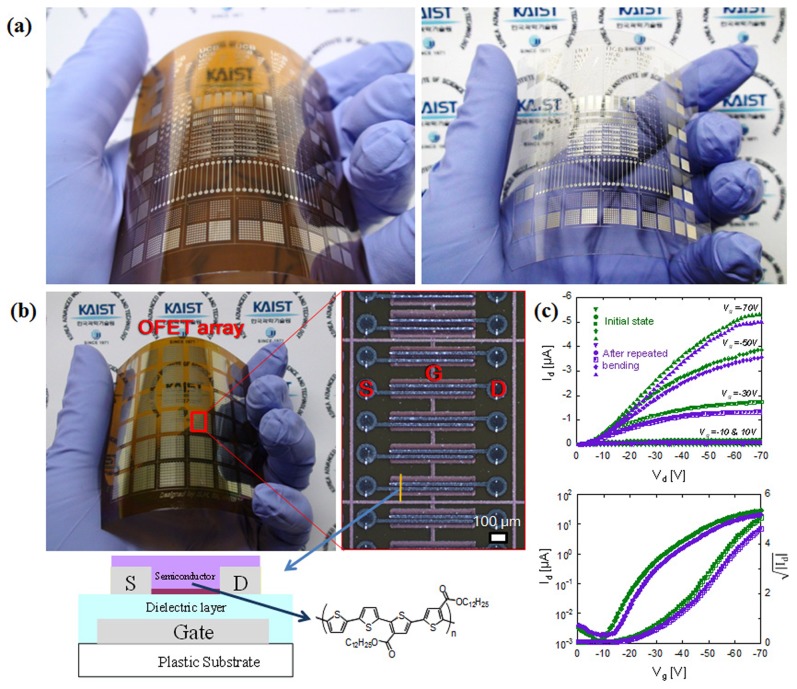
Large Area Flexible Electronics Demonstration through DDMP Process. a, Single layer Ag micro patterns on PI(left) and PET(right) flexible substrates (4 inch wafer size). The pattern size varies from several microns to several centimeters. **b,** Multiple layer Ag micro patterning for OFET array demonstration on a flexible substrate. Right picture shows the magnified view of OFET arrays. Bottom picture shows the semiconducting polymer and cross sectional structure of the OFET. Letters *“G”,”S”,”D”* represent gate, source and drain respectively. **c,** Output (top graph) and transfer (bottom graph) characteristics of OFET before (green) and after (purple) cyclic bending test. Note that no significant OFET performance change was observed after 100,000 times bending.

The elimination of vacuum requirement and photo-masks removes substrate size restriction and enables current DDMP process to be readily applied for large area electronics fabrication. The substantial cost for vacuum chamber and photo-mask especially for large substrate render conventional vacuum deposition methods less attractive. Other major advantage of DDMP process is the low temperature process which can be applied for direct metal patterning on the heat sensitive polymer substrate without damaging. In contrast, conventional metal patterning methods usually apply high temperature metal evaporation, which easily induce polymer substrate damage. Figure 4a shows Ag micro patterns on PI (left) and transparent PET (right) flexible 4 inch substrates. The pattern size varies from several microns to several centimeters. Besides the cost effectiveness, DDMP process is much faster with higher process flexibility than conventional process. The metal patterning on 4 inch PET substrate (figure 4a, right picture) by scanning 320 mW laser at 1 m/s speed was prepared in only 30 seconds (See the real time Movie clip S2 for DDMP process demonstration), which will take at least several hours to several days by conventional vacuum deposition and photo-lithography process for a single item fabrication. This ultra-fast metal patterning was made possible because DDMP process needs very small laser power thus the fast laser scanning speed can be applied due to very strong laser absorption and melting temperature drop of the Ag NPs. Moreover, DDMP process is digital in nature and enables real time metal pattern design change by just simply modification of the computer data. However, conventional expensive photomask needs to be fabricated again once the design is changed. Combining these advantages, the DDMP process can reduce the process time dramatically and further this will lead to cost-reduction. Electronics industry used to mass produce small variety items. However, in these days, paradigm is shifting from large volume, small variety production to small volume, large variety item production. Fast and digital process of DDMP is very important especially for small volume, large variety and rapid turnaround product.

To realize more sophisticated electronics, DDMP process can be applied for multilayered structures by repeating the unit process. Figure 4b shows the 11,520 organic field effect transistor (OFET) arrays fabricated on a PI substrate. The OFETs fabricated in this work have a typical bottom gate/bottom contact coplanar transistor configuration (figure 4b, bottom picture) wherein the channel length is defined by the separation between the two parallel electrodes (source and drain) on top of poly-vinyl-phenol (PVP) dielectric layer on gate electrodes. Carboxylate-functionalized polythiophene (figure 4b, inset of bottom picture) with increased air stability [30] was synthesized, dissolved in warm (>45°C) 1,2-dichlorobenzene (*o*-DCB) solvent (3 mg/mL) and spincoated as an active layer. The detailed information on the OFET and semoconducting polymer can be found in File S1. OFETs with various channel lengths (2∼50 µm) and 650 µm channel width were fabricated (figure 4b right picture). The OFET output and transfer characteristics are shown in figure 4c for 6 µm channel length. The OFET shows typical output and transfer characteristics with operation in p-type accumulation mode with I_on_/I_off_ ratio of 10^3^∼10^4^ and threshold voltage (V_t_) of −25 V. The mobility extracted from the saturated transfer characteristics of the OFET was found to be around 0.001∼0.002 cm^2^/V·s. Due to the flexibility of the polymer substrate, the devices are easily subject to a variety of mechanical deformation conditions. High performance flexible electronics are supposed to maintain its functionality under various mechanical deformations. The transistor performance characteristics were measured before and after the 100,000 times bending cycles. figure 4c shows output characteristics (top graph) and transfer characteristics (bottom graph) of OFETs on polyimide substrate before cyclic loading (green) and after 20 k bending cycles (purple) for a 6 µm channel. No significant changes in the OFET performance were observed except slight drop in drain current probably due to the semiconductor polymer degradation during the high voltage measurement. Similar OFET performance signifies that the electrical performance of the devices fabricated by the current process is reasonably good for application to flexible electronics.

Above demonstrations signified that DDMP processing for flexible metal electrode fabrication is a promising approach in countless applications such as high performance flexible opto-electronics (solar cell, display, touch panel etc.), wearable computer, portable flexible electronics in a simple, fast, low temperature, non-vacuum, scalable and cost-effective way.

## Conclusions

In summary, we have successfully demonstrated that the direct digital metal patterning on a flexible polymer substrate by applying CW laser on solution deposited Ag NP ink film. This new metal patterning method enables simple, direct, low temperature, high resolution, digital metal patterning without need for any conventional vacuum metal deposition and photomask processes. The DDMP process was successfully applied to demonstrate low resistivity Ag patterning (2.1 µΩ·cm) and the metal electrode fabrication for working flexible electronics devices such as high performance OFET arrays on a polymer substrate. The remarkable new digital direct metal patterning could be achieved due to the combination of the laser technology and the novel thermal characteristics of the metal NPs (reduced melting temperature and strong laser absorption). The DDMP process can produce high resolution (down to few microns resolution) metal patterning on a large polymer substrate (over 4 inch wafer) in a very fast single step (fraction of minutes). Moreover, the *“digital”* nature of our process can remove the need for the expensive photo-mask and allows easy design change. All these characteristics are expected to greatly contribute to the development of next generation cost-effective metal patterning realizing flexible electronics. In this manner, our DDMP process developed in this work enables flexible electronic circuits to be mounted at non-flat surfaces where to date we have been unable to provide electrical and mechanical functionalities. We believe that our research suggests an important step toward producing intelligent and multifunctional flexible electric devices as friendly human/electronics interface, and ultimately contribute to the applications in ubiquitous electronics.

## Supporting Information

Figure S1 **DDMP process experiment set-up.** After coating Ag NP ink on the substrate, continuous wave green wavelength lasers (Nd:YAG-532 nm, Ar ion-514.5 nm) were scanned by 2D galvanometric scanning mirror system (SCANLAB, hyrrySCAN II) to raster scan focused laser spot and induce local Ag NP melting. The laser scanner system was controlled by computer with CAD software (SCAPS GmbH, SAMLight) to draw arbitrary 2D images. The laser scanning speed and laser power were adjusted in 0∼3 m/s and 0∼400 mW depending on the substrate.
(TIF)

Figure S2 **Laser power and laser scanning speed values for combinatorial study for** **Figure 2a** **.** Regular laser power range was 5∼160 mW and 2× laser power range was 10∼320 mW. Regular laser power was applied to PI (top left), Glass (top right) and PET (bottom right) and 2× laser power was applied to PI-2x power (bottom left) in Figure 2a. The total sample size for combinatorial study was 2 cm×2 cm and the small unit square for each laser power and laser scanning speed was 1 mm×1 mm.
(TIF)

Figure S3 **Experimental setup for Ag NP laser sintering time characterization in** **Figure 2c** **.** The time lag between laser irradiation (green line) and conductive metal electrode formation (purple line) was measured by transient resistance change during the laser irradiation on Ag NP ink. The voltage signals were recorded with oscilloscope (Agilent, InfinniVision). The laser irradiation time was controlled by acousto optic modulator (AOM) connected to delay generator (Stanford Research Systems, DG535).
(TIF)

Figure S4 **Semiconducting Polymer Synthesis.**
(TIF)

Movie clip S1 **Cyclic Bending Test Video Clip of DDMP processed Ag electrode pattern on a PI substrate.**
(AVI)

Movie clip S2 **Real Time Video Clip of DDMP (Digital Direct Metal Patterning) on a 4 inch PET substrate.** Fume was generated due to the decomposition of SAM and removed from the laser spot by suction during the process.
(AVI)

File S1 **Supporting Information.**
(DOCX)
