# Supplementary figures and images for: Next Generation Non-Vacuum, Maskless, Low Temperature Nanoparticle Ink Laser Digital Direct Metal Patterning for a Large Area Flexible Electronics
Source: PLoS One. 2012 Aug 10;7(8):e42315. doi: 10.1371/journal.pone.0042315 (PMC3416833; doi:10.1371/journal.pone.0042315)

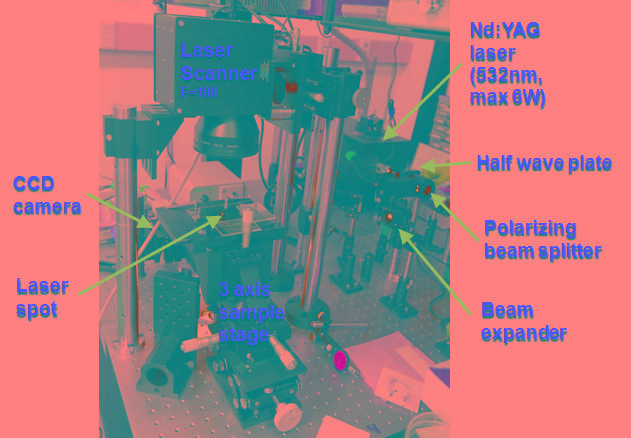

Supplement: Figure S1 — DDMP process experiment set-up. After coating Ag NP ink on the substrate, continuous wave green wavelength lasers (Nd:YAG-532 nm, Ar ion-514.5 nm) were scanned by 2D galvanometric scanning mirror system (SCANLAB, hyrrySCAN II) to raster scan focused laser spot and induce local Ag NP melting. The laser scanner system was controlled by computer with CAD software (SCAPS GmbH, SAMLight) to draw arbitrary 2D images. The laser scanning speed and laser power were adjusted in 0∼3 m/s and 0∼400 mW depending on the substrate. (TIF) [file pone.0042315.s001.tif]

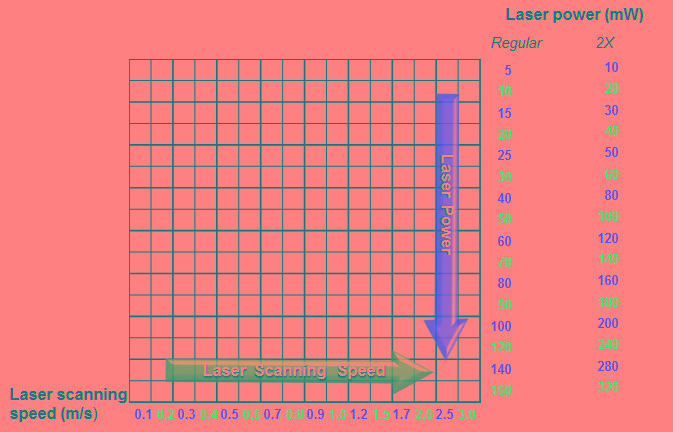

Supplement: Figure S2 — Laser power and laser scanning speed values for combinatorial study for Figure 2a . Regular laser power range was 5∼160 mW and 2× laser power range was 10∼320 mW. Regular laser power was applied to PI (top left), Glass (top right) and PET (bottom right) and 2× laser power was applied to PI-2x power (bottom left) in Figure 2a. The total sample size for combinatorial study was 2 cm×2 cm and the small unit square for each laser power and laser scanning speed was 1 mm×1 mm. (TIF) [file pone.0042315.s002.tif]

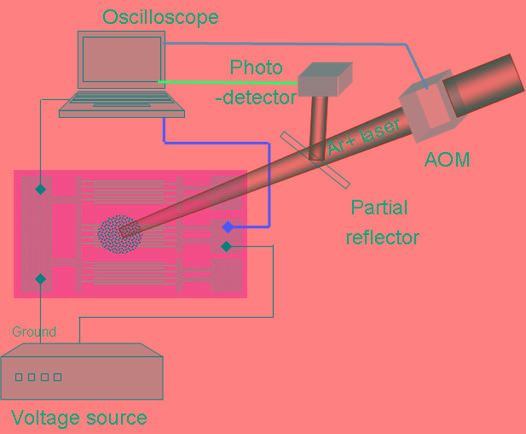

Supplement: Figure S3 — Experimental setup for Ag NP laser sintering time characterization in Figure 2c . The time lag between laser irradiation (green line) and conductive metal electrode formation (purple line) was measured by transient resistance change during the laser irradiation on Ag NP ink. The voltage signals were recorded with oscilloscope (Agilent, InfinniVision). The laser irradiation time was controlled by acousto optic modulator (AOM) connected to delay generator (Stanford Research Systems, DG535). (TIF) [file pone.0042315.s003.tif]

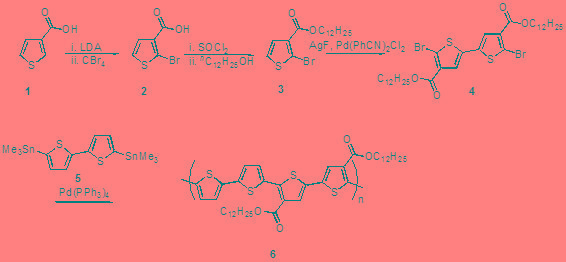

Supplement: Figure S4 — Semiconducting Polymer Synthesis. (TIF) [file pone.0042315.s004.tif]
