# Supplementary material for: Next Generation Non-Vacuum, Maskless, Low Temperature Nanoparticle Ink Laser Digital Direct Metal Patterning for a Large Area Flexible Electronics
Source: PLoS One. 2012 Aug 10;7(8):e42315. doi: 10.1371/journal.pone.0042315 (PMC3416833; doi:10.1371/journal.pone.0042315)
Supplement: File S1 — Supporting Information. (DOCX) [file pone.0042315.s007.docx]

**Supporting Information**

Next Generation Non-vacuum, Maskless, Low Temperature Nanoparticle Ink Laser Digital Direct Metal Patterning for a Large Area Flexible Electronics

*Junyeob Yeo^1^, Sukjoon Hong^1^, Daehoo Lee^2^, Nico Hotz^3^, Ming-Tsang Lee^4^, Costas P. Grigoropoulos^2^†, Seung Hwan Ko^1^†*.

†correspondence to: [maxko@kaist.ac.kr](mailto:maxko@kaist.ac.kr), [cgrigoro@me.berkeley.edu](mailto:cgrigoro@me.berkeley.edu)

**1. DDMP process set up**

**
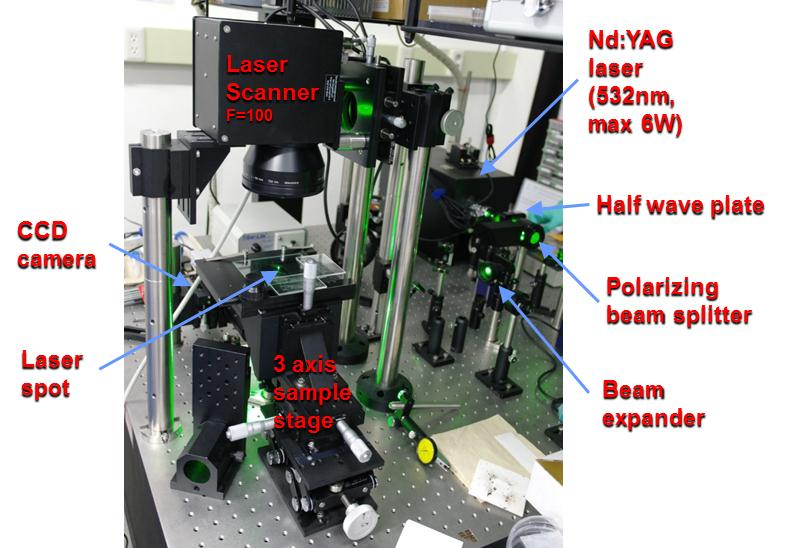
**

**Figure S1**. **DDMP process experiment set-up.** After coating Ag NP ink on the substrate, continuous wave green wavelength lasers (Nd:YAG-532nm, Ar ion-514.5nm) were scanned by 2D galvanometric scanning mirror system (SCANLAB, hyrrySCAN II) to raster scan focused laser spot and induce local Ag NP melting. The laser scanner system was controlled by computer with CAD software (SCAPS GmbH, SAMLight) to draw arbitrary 2D images. The laser scanning speed and laser power were adjusted in 0~3 m/s and 0~400 mW depending on the substrate.

**2. Ag NP laser processing condition characterization**


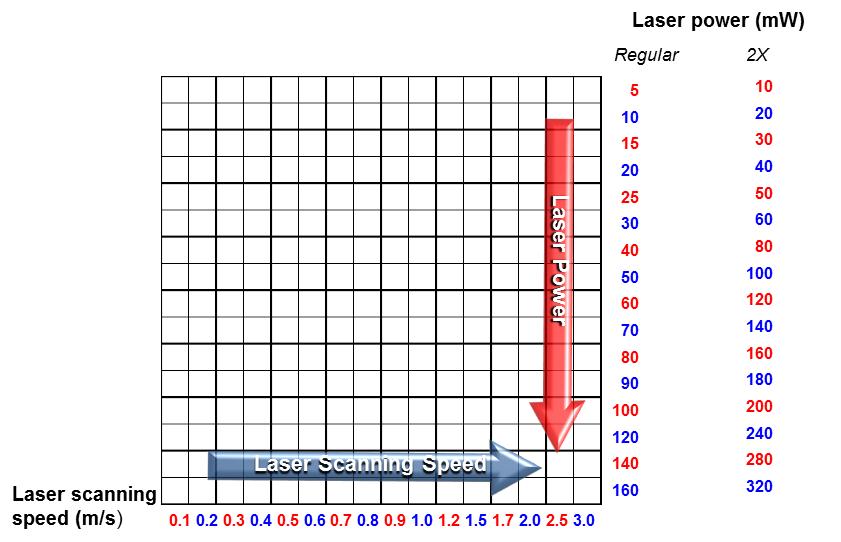


**Figure S2**. **Laser power and laser scanning speed values for combinatorial study for Figure 2a** Regular laser power range was 5~160 mW and 2X laser power range was 10~320 mW. Regular laser power was applied to PI (top left), Glass (top right) and PET (bottom right) and 2X laser power was applied to PI-2x power (bottom left) in Figure 2a. The total sample size for combinatorial study was 2cm × 2cm and the small unit square for each laser power and laser scanning speed was 1mm × 1mm.

**3. Ag NP laser processing time characterization**


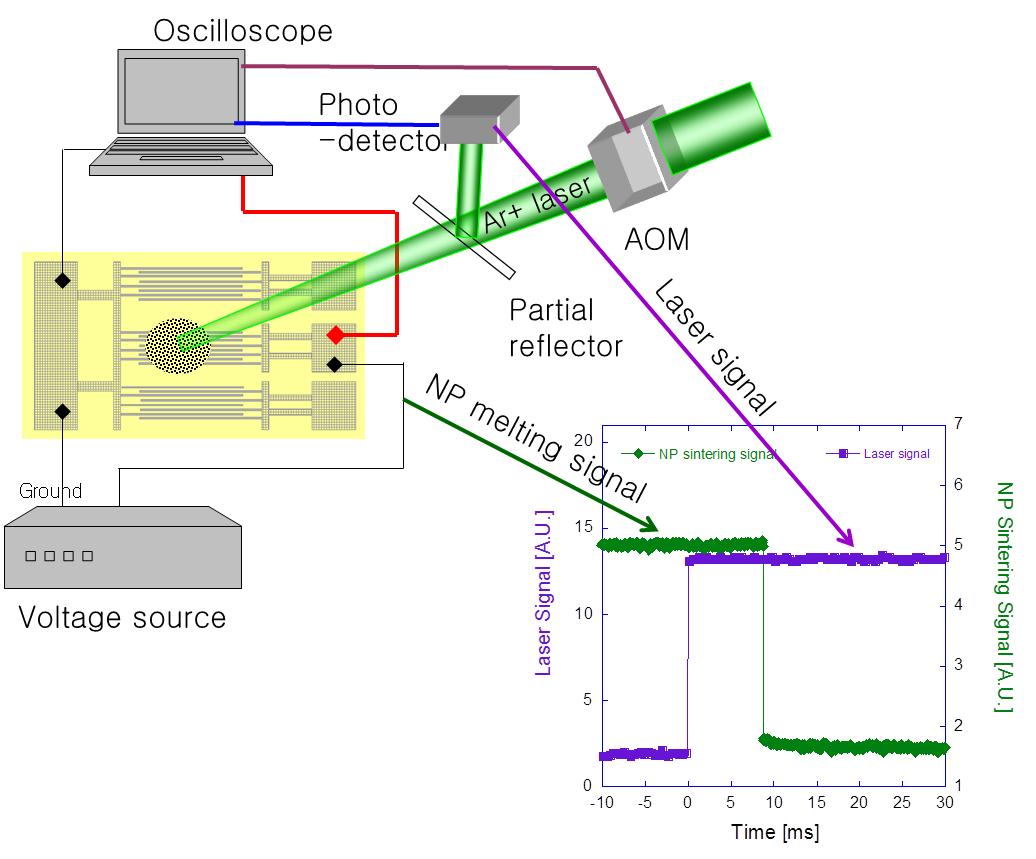


**Figure S3**. **Experimental setup for Ag NP laser sintering time characterization in Figure 2c** The time lag between laser irradiation (green line) and conductive metal electrode formation (purple line) was measured by transient resistance change during the laser irradiation on Ag NP ink. The voltage signals were recorded with oscilloscope (Agilent, InfinniVision). The laser irradiation time was controlled by acousto optic modulator (AOM) connected to delay generator (Stanford Research Systems, DG535).

**4. Electrical characterization**

**Resistivity measurement**: The resistivity was measured using a probe station and an atomic force microscopy (AFM). Line-shaped metal conductors with the length (*L*) of 180 μm between two electrode pads (40 μm x 40 μm) were simultaneously fabricated by the DDMP process. The resistance (*R*) was measured on a probe station, and the cross-sectional area (*A*) of the silver line was measured by AFM. The resistivity (*ρ*) was calculated from the equation *ρ=R·A/L* in which R is resistance, A is the cross sectional area of the silver nano/micro line, and L is the length of the test sample. R was measured on a probe station (MST5000A, MS Tech, Korea) with 4-axis micro manipulators having gold coated probe tip (10 μm tip diameter) in a dark Faraday cage using semiconductor analyzer (HP4145B, Hewlett-Packard, USA). Values for *A* and *L* were measured from AFM scanning data (NANOMAN, Veeco, USA) and from optical microscope images, respectively.

**5. Semiconducting polymer synthesis and characterization**

**Materials**: All chemicals were purchased from Aldrich and used without further purification unless otherwise noted. All solvents were purified on a solvent purification system. All reactions were performed under N_2_ unless otherwise noted. All extracts were dried over anhydrous MgSO_4_ and solvents were removed by rotary evaporation with vacuum assist. Flash chromatography was performed using Merck Kieselgel60 (230 - 400 mesh) silica.

**Characterization**: ^1^H NMR spectra were recorded with Brüker AMX-300, AM-400 or DRX-500 instruments using CDCl_3_ as the solvent unless otherwise noted. Analytical size exclusion chromatography (SEC) in THF was performed at 35 °C at a nominal flow rate of a 1.0 mL/min on a chromatography line calibrated with linear polystyrene standards (162 – 2,100,000 Da) and fitted with three 7.5 × 300 mm PL gel columns (5 μm particle size). The columns have a pore size of 105, 103, and 500 Å, respectively. The SEC system consists of a Waters 510 pump, a Waters 717 autosampler, and a Waters 486 UV-Vis detector detecting at 254 and 450 nm. Polymer solutions were prepared by adding 100 μL of a 1 mg/mL solution of the polymer in *o*-DCB to 1 mL of THF, then filtering through 200 nm pore size PVDF filters (Whatman) before injection.

**Figure S4.** Semiconducting Polymer Synthesis.

2-Bromothiophene-3-carboxlic acid (2) was prepared from thiophene-3-carboxylic acid 1 using a literature procedure [30]. ^1^H NMR: 7.25 (1H, d, *J* = 6 Hz), 7.45 (1H, d, *J* = 6 Hz). Dodecyl 2-bromothiophene-3-carboxylate (3) was prepared from carboxylic acid using a published procedure [30]. ^1^H NMR:  7.36 (1H, d, *J* = 6 Hz), 7.20 (1H, d, *J* = 6 Hz), 4.26 (2H, t, *J* = 6 Hz), 1.71-1.79 (2H, m), 1.39-1.43 (2H, m), 1.19-1.38 (16H, m), 0.86 (3H, t, *J* = 6 Hz). Didodecyl 5,5’-dibromo-2,2’-bithiophene-4,4’-dicarboxylate (4) was prepared using a published procedure [30]. ^1^H NMR:  7.36 (2H, s), 4.30 (4H, t, *J* = 6 Hz), 1.71-1.79 (4H, m), 1.20-1.50 (36H, m), 0.89 (6H, t, *J* = 6 Hz). 5,5’-Bis(trimethylstannyl)2,2’-bithiophene (5) was synthesized using a published procedure [30]. ^1^H NMR: 7.27 (2H, d, *J* = 3 Hz), 7.11 (2H, d, *J* = 3 Hz), 0.39 (18H, s). Poly(didodecyl 2,2’-bithiophene-4,4’-dicarboxylate-*co*-2,2’-bithiophene) (6) was synthesized according a published procedure [30]. ^1^H NMR (*o-*DCB-d_4_):  7.84 (1H, s), 7.75 (1H, d), 7.36 (1H, d), 4.55 (2H, t, *J* = 6 Hz), 1.97 (2H, t, *J* = 6 Hz), 1.60 (2H, quintet, *J* = 7 Hz), 1.30-1.60 (18H, m), 1.07 (3H, t, *J* = 6 Hz). THF-SEC: M_w_ = 6.7 kDa, PDI = 1.4.

**Supporting Information Legend**

**Figure S1**. **DDMP process experiment set-up.** After coating Ag NP ink on the substrate, continuous wave green wavelength lasers (Nd:YAG-532nm, Ar ion-514.5nm) were scanned by 2D galvanometric scanning mirror system (SCANLAB, hyrrySCAN II) to raster scan focused laser spot and induce local Ag NP melting. The laser scanner system was controlled by computer with CAD software (SCAPS GmbH, SAMLight) to draw arbitrary 2D images. The laser scanning speed and laser power were adjusted in 0~3 m/s and 0~400 mW depending on the substrate.

**Figure S2**. **Laser power and laser scanning speed values for combinatorial study for Figure 2a** Regular laser power range was 5~160 mW and 2X laser power range was 10~320 mW. Regular laser power was applied to PI (top left), Glass (top right) and PET (bottom right) and 2X laser power was applied to PI-2x power (bottom left) in Figure 2a. The total sample size for combinatorial study was 2cm × 2cm and the small unit square for each laser power and laser scanning speed was 1mm × 1mm.

**Figure S3**. **Experimental setup for Ag NP laser sintering time characterization in Figure 2c** The time lag between laser irradiation (green line) and conductive metal electrode formation (purple line) was measured by transient resistance change during the laser irradiation on Ag NP ink. The voltage signals were recorded with oscilloscope (Agilent, InfinniVision). The laser irradiation time was controlled by acousto optic modulator (AOM) connected to delay generator (Stanford Research Systems, DG535).

**Figure S4.** Semiconducting Polymer Synthesis

**Movie Clip**

Movie clip S1) **Cyclic Bending Test Video Clip of DDMP processed Ag electrode pattern on a PI substrate.**

Movie clip S2) **Real Time Video Clip of DDMP (Digital Direct Metal Patterning) on a 4 inch PET substrate**.

Fume was generated due to the decomposition of SAM and removed from the laser spot by suction during the process.
